# Effects of Thermoforming Parameters on Woven Carbon Fiber Thermoplastic Composites

**DOI:** 10.3390/ma17163932

**Published:** 2024-08-07

**Authors:** Shun-Fa Hwang, Cheng-Yi Yang, Shao-Hao Huang

**Affiliations:** Department of Mechanical Engineering, National Yunlin University of Science and Technology, 123 University Road, Sec. 3, Yunlin 64002, Taiwan; b10611117@yuntech.edu.tw (C.-Y.Y.); m10611063@yuntech.edu.tw (S.-H.H.)

**Keywords:** thermoforming, thermoplastic composite, finite element analysis, Taguchi orthogonal array, process parameter

## Abstract

The quality of woven carbon fiber fabric/polycarbonate thermoplastic composites after thermoforming and demolding was investigated using finite element simulation and the Taguchi orthogonal array. The simulation utilized a discrete approach with a micro-mechanical model to describe the deformation of woven carbon fabric, combined with a resin model. This simulation was validated with bias extension tests at five temperatures. The thermoforming process parameters considered were blank temperature, mold temperature, and blank holding pressure, with three levels for each factor. Optimal values for the fiber-enclosed angle, spring-back angle, mold shape fitness, and the strain of the U-shaped workpiece were desired. The results indicated that the comparison of the stress-displacement curve of bias extension tests verified the application of the discrete finite element method. Results from the Taguchi array indicated that blank holding pressure was the dominant parameter, with the optimal value being 1.18 kPa. Blank temperature was the second most significant factor, effective in the range of 160 °C to 230 °C, while mold temperature had a minor effect. Furthermore, the four quality values are dependent and have a similar trend. The best combination was identified as a blank holding press of 1.18 kPa, a blank temperature of 230 °C, and a mold temperature of 190 °C.

## 1. Introduction

Composite materials are competitive candidates for weight-saving applications in the automobile, sports, and aerospace industries. While thermoset composites are more popular due to their mechanical properties, thermoplastic composites have gained attention for their recyclability, particularly in the automotive industry [1,2]. Unlike thermoset structures that use prepregs, semi-finished products like sheets are used to manufacture thermoplastic composite products to avoid lay-up issues. Forming processes such as hydroforming, rubber pad forming, and thermoforming are simple and fast [3,4], with thermoforming being especially attractive for complex shapes and automation [5].

In the thermoforming process, semi-finished sheets are softened by heat before pressing. The pressing temperature is generally above the glass transition or melting temperature, affecting the surface quality of the product. After pressing, the temperature is reduced to an appropriate value, and the product is demolded. For instance, Behrens et al. [6] manufactured a down-scaled battery tray for a plug-in-hybrid vehicle from woven glass fabric/polyamide 6 thermoplastic blanks via an automated thermoforming process at 260 °C. A hat profile structure was also produced from the same blanks at 270 °C, followed by bonding a plate to create a crash box [7]. A semi-finished thermoplastic tube was fabricated into a shaft by Maron et al. [8] using two thermoforming steps and local heating above the melting point.

The product quality in thermoforming depends on parameters such as stacking sequence, blank temperature, mold temperature, blank holding pressure, cooling rate, and forming rate [9,10,11]. Han et al. [12] investigated the appropriate molding temperature from the final dimensions of V-shaped parts thermoformed from carbon fiber/polyphenylene sulfide sheets. Yin et al. [13] experimentally concluded that thermoplastic type, fiber orientation, and mold fixing methods are crucial for producing double-curvature thermoplastic composite parts. The effect of the stacking sequence was also considered to avoid wrinkling in glass fiber-reinforced polypropylene sheets during drawing [14]. Lee et al. [15] measured the mechanical properties of glass fiber-reinforced polypropylene composite parts after thermoforming to study the effect of cooling rate. Overall, thermoforming is a complex process with multiple variables, making it challenging to select appropriate process parameters.

The finite element method (FEM) is suitable for investigating thermoforming process parameters for thermoplastic composites due to the difficulty in obtaining an analytic model. The FEM for this topic can be categorized into continuous, semi-discrete, and discrete approaches. In the continuous approach, composite materials are meshed with membrane or shell elements using continuous macroscopic models, simulating shear and bending deformation but neglecting fiber sliding and rotation [16,17,18,19,20]. The discrete approach [21,22,23] addresses micro-mechanical behaviors like the trellis mechanism, fiber reorientation, and viscoelasticity, combining fiber and resin models to represent thermoplastic materials during the thermoforming process. While this approach accurately illustrates the warp and weft movement and large deformation, as well as damage behaviors, it requires many properties and parameters of fibers and resins, which may be difficult to obtain. The semi-discrete approach [24,25] combines the advantages of both continuous and discrete approaches but requires special continuous elements affected by micro-mechanical behavior.

To fully explore the interaction of weft and warp yarns, the discrete approach is commonly used to simulate thermoforming behavior. For example, De Luycher et al. [26] created a unique hexahedral element with a hyperelastic model to portray segment yarns’ transverse behaviors. Charmetant et al. [27] proposed a hyperelastic model for woven fiber fabric under extension loading, which was further verified by biaxial tension and shear tests. Sidhu et al. [28] developed a new finite element approach for stamping analysis of plain-weave textile composite preforms using 3D truss and shell elements. Tabiei and Murugesan [21] presented a micro-mechanical model of loosely woven fabric and a resin model to simulate the thermoforming of woven fabric composite materials. Hwang and Yu [29] used the discrete finite element method to reasonably predict the spring-in behavior of woven carbon fiber fabric/polycarbonate thermoplastic composite sheets.

This study investigates thermoforming parameter effects for woven carbon fiber fabric/polycarbonate composites using finite element simulation and the Taguchi orthogonal array. The thermoplastic composite blank was deformed into a U-shaped structure. Differential scanning calorimetry and dynamic mechanical analysis measured temperature-related composite properties. The finite element method was validated by comparing stress-displacement curves from bias extension tests at five temperatures. Process parameters—blank temperature, mold temperature, and blank holding pressure—were analyzed using the Taguchi array with three factors and three levels. The objectives included optimizing the fiber-included angle, part-mold shape fitness, spring-back angle, and part strain under extension. Optimal process parameter levels were identified based on these objectives.

## 2. Thermoplastic Composite Material and Thermoforming Process

**Figure 1:**
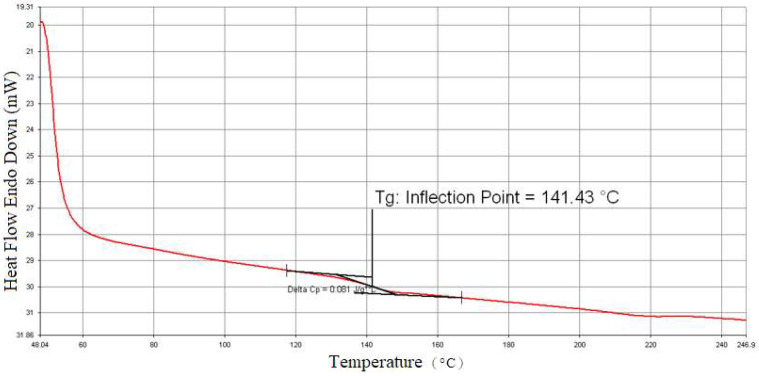
The measured results from differential scanning calorimeter.

**Figure 2:**
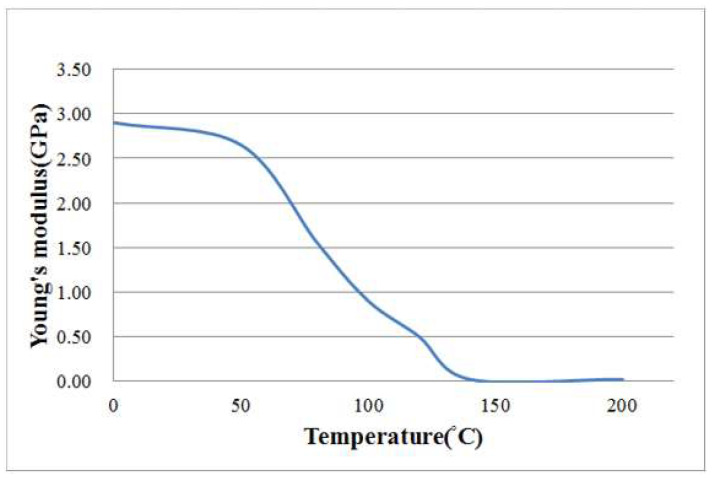
Variation of Young’s modulus with temperature for polycarbonate.

Thermoplastic composite blanks from Formosa Plastics Corporation consisted of polycarbonate resin and plain-woven carbon fabric made from TAIRYFIL TC-300 3k-carbon fiber yarns. Differential scanning calorimetry tested the thermoplastic blank for thermodynamic properties at a 5 °C/min temperature increase rate, revealing a glass transition temperature around 141.4 °C as shown in Figure 1. Even though it is not clear in Figure 1, the melting temperature is about 230 °C, as judged from more examples. Additionally, dynamic mechanical analysis was employed to assess the Young’s modulus of the composite blank at a temperature increase rate of 2 °C/min. Substracting the effects of carbon fibers from the composite, the relationship between Young’s modulus and temperature for polycarbonate was determined and is depicted in Figure 2.

**Figure 3:**
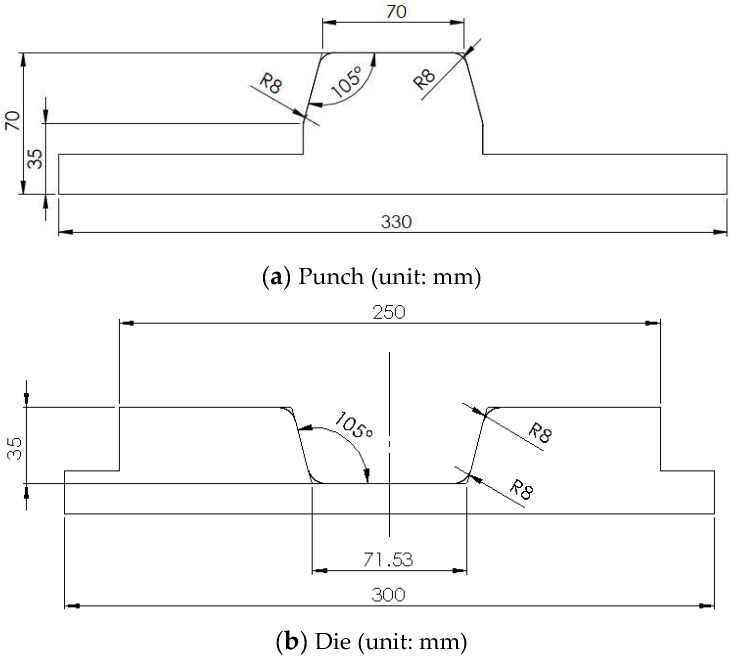
Dimensions of punch and die.

In this study, the aforementioned composite blanks with dimensions of 220 × 120 × 1.2 mm were thermoformed into U-shaped structures. The stacking sequence of these blanks was [(+45°/−45°)]_6_, indicating that each composite fabric layer had fiber angles of +45° and −45°, with a total of 6 layers of fiber fabric. The detailed dimensions of the punch and die are provided in Figure 3a,b. An infrared heater was used to heat the composite blank, which was placed on the lower mold, to the desired temperature. The mold temperature was controlled by heaters buried in the mold. Subsequently, a forming rate of 25 mm/s was applied, and a final forming pressure of 4.9 Pa (50 kg/cm^2^) was maintained. During the forming process, a blank holding pressure was applied. After forming, the thermoplastic composite product was naturally cooled to 25 °C before being demolded. The thermoforming process parameters investigated included blank temperature, mold temperature, and blank holding pressure.

## 3. Finite Element Simulation

**Figure 4:**
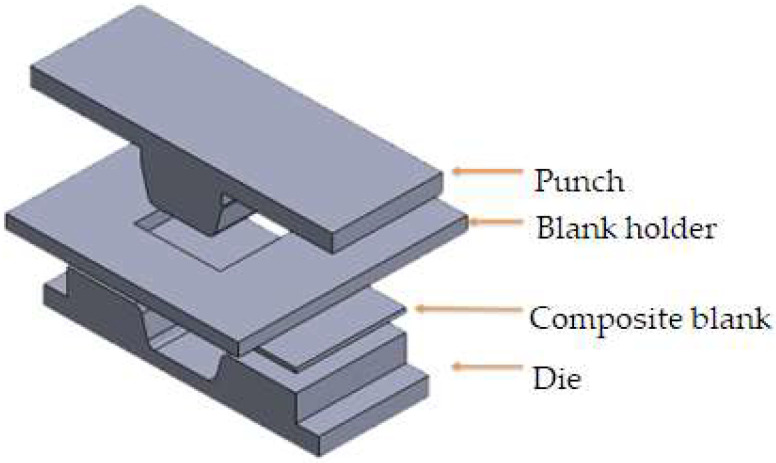
The model of the thermoforming process.

A commercial finite element software, LS-DYNA R10.0, was utilized to simulate the thermoforming processes in explicit mode, and the model is illustrated in Figure 4. The discrete approach of the finite element method was chosen to describe the deformed behavior and fiber angle changes between the weft and warp yarns. Tabiel et al. [21] implemented a material card using this discrete approach, leading to the creation of MAT 234 in LS-DYNA. This micro-mechanical material model included the locking situation between the two yarns, their reorientation, and fiber crimping. Numerous carbon fiber parameters were required for this discrete approach, with the main parameters listed in Table 1. To simulate the thermoplastic resin, a standard material model, MAT 004, was applied, which is suitable for elasticity, plasticity, and thermal analysis. In this study, polycarbonate was treated as an elastic material with consolidation effects, and the variation of Young’s modulus with temperature is shown in Figure 2. Additional properties of the polycarbonate used in the simulation are provided in Table 2.

**Figure 5:**
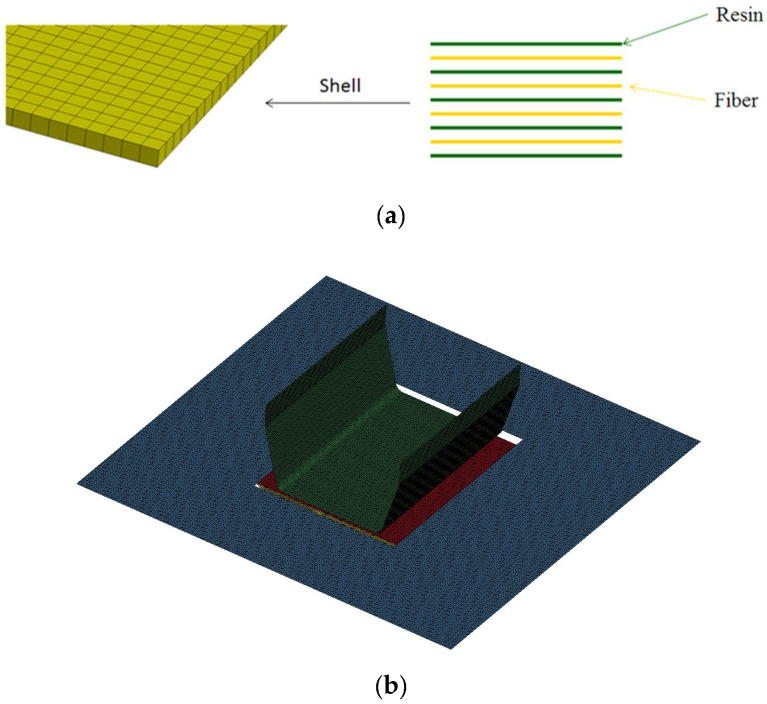
The element and meshes for the U-shaped structure. (**a**) The four-fiber layer and five-resin layer included in one shell element; (**b**) Finite element mesh for the U-shaped structure.

Although the thermoplastic composite blank consisted of six fiber fabric layers and polycarbonate, it was modeled as a single shell element. In the discrete approach, each shell element comprised four fiber fabric layers and five resin layers, interlaced alternately as shown in Figure 5a. The thickness of each fabric and resin layer was calculated from the 1.2 mm total thickness of the composite blank and the 0.3 fiber volume fraction, resulting in 0.09 mm for each fabric layer and 0.168 mm for each resin layer. Using these nine layers to represent the composite blank was proven to yield the best simulation results compared to other layer number combinations [30]. In this simulation, the shell element for the blank had a size of 1 × 1 mm. Similarly, 1 × 1 mm shell elements were used for the punch, die, and blank holder, as shown in Figure 5b. Although a Young’s modulus of 210 GPa, a Poisson’s ratio of 0.3, and a density of 7.83 g/cm^3^ were assigned, these three parts were treated as rigid bodies.

**Figure 6:**
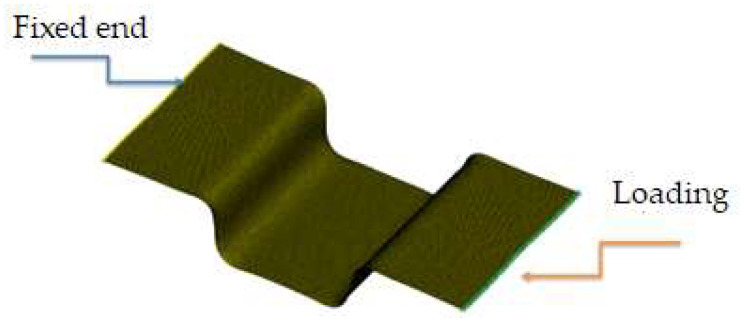
Loading conditions of the U-shaped structure after demolding.

The Taguchi method is an orthogonal array technique that can identify optimal combinations of factor levels with fewer trials. In this study, the Taguchi array was used to investigate the effects of blank temperature, mold temperature, and blank holding pressure. For these three factors, three levels were chosen, and the L_9_(3^3^) array was employed to analyze their effects rather than to find an optimal combination. As shown in Table 3, both the blank temperature and mold temperature had three levels ranging from 160 °C to 230 °C. The temperature of 160 °C is above the glass transition temperature, and 230 °C is near the melting temperature. The blank holding pressure ranged from zero to 2.36 kPa to observe its effect. In the simulation for the forming process, heat conduction occurred through transient analysis due to varying temperatures in different parts. After forming, all four parts shown in Figure 4 were cooled to room temperature. The demolding process was performed to analyze the spring-back behavior of the workpiece. Unlike metals, explicit mode was used for this thermoplastic composite material because the MAT 234 material card cannot be used in implicit mode. After the spring-back analysis, the workpiece quality was evaluated based on the fiber-enclosed angle, the spring-back angle, and the fitness of its shape with the mold. Additionally, the demolded workpiece was subjected to a uniaxial tensile loading of 50 MPa, as shown in Figure 6, to obtain the strain. Consequently, there were three consecutive stages of finite element analysis: thermoforming, demolding, and extension.

## 4. Results and Discussion

### 4.1. Bias Extension Test

**Figure 7:**
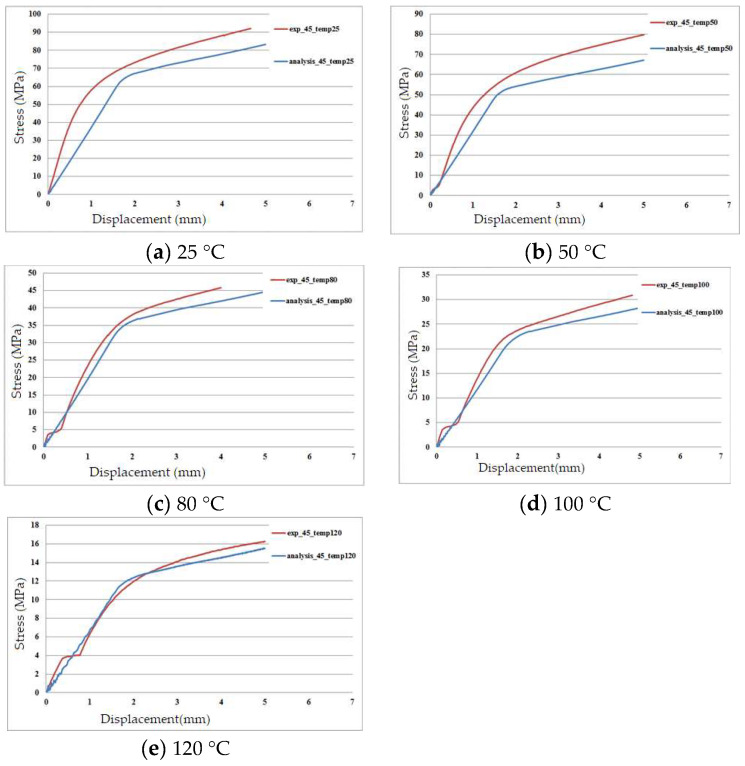
Stress-displacement curves of bias extension tests under five temperatures.

Bias extension tests were conducted both experimentally and numerically to compare the stress-displacement curves of the carbon fiber fabric/polycarbonate thermoplastic composites. The bias extension test is a uniaxial tension test where the fiber angles are ±45 degrees, generating shear stress in the central part of the specimen. In this study, these tests were used to validate the finite element analysis at different temperatures. The stacking sequence of the thermoplastic composite specimens was [(+45°/−45°)]_6_, and they were tested using an Instron 5982 with a loading rate of 2 mm/min. By utilizing a cabinet with a constant temperature, the testing temperature was controlled at 25 °C, 50 °C, 80 °C, 100 °C, and 120 °C. Similar to the aforementioned finite element model, shell elements with nine layers and a size of 1 × 1 mm were used to represent the composite specimen. Additionally, end tabs were bonded to the specimen and simulated with 1 × 1 × 1 mm solid elements. The comparison of the stress-displacement curves obtained from experiments and simulations is shown in Figure 7a–e. Due to varying temperatures, different Young’s moduli for the resin were used in the finite element analysis, according to Figure 2. From these figures, the differences between the experimental and simulation results at 25 °C and 50 °C are slightly larger but still within a reasonable range. Overall, the stress-displacement behavior of the thermoplastic composite under different temperatures is well simulated by the present finite element method.

### 4.2. Results from the Taguchi Orthogonal Array

In this study, the primary focus of the finite element simulation was on the thermoforming and demolding processes. After these two stages of simulation, the quality of the workpiece was evaluated by measuring the fiber-enclosed angle, the spring-back angle, and the fitness under different combinations of processing parameters. This two-stage finite element analysis, which combines structural analysis with transient thermal analysis, has been verified by Hwang et al. [29] for assessing the spring-in angle of the same thermoplastic composite thermoformed into a V-shaped structure.

**Figure 8:**
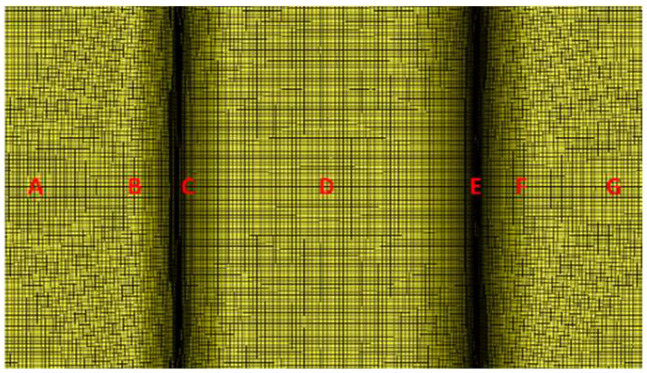
Locations A–G to measure the fiber-enclosed angle.

Before thermoforming, the fiber-enclosed angle between the warp and the weft of the blank was 90° because it was a woven fabric. After thermoforming, this angle changes, potentially affecting the performance of the formed structure. One advantage of the discrete approach is that it illustrates the fiber-enclosed angle throughout the entire simulation process. As shown in Figure 8, the fiber-enclosed angles at 7 points along the length of the U-shaped workpiece are measured. The fiber-enclosed angles obtained from different combinations of process parameters are listed in Table 4. The desired fiber-enclosed angle is 90°; deviations from this value indicate a suboptimal combination of process parameters. From this table, it can be seen that the fiber-enclosed angles for EX-1, EX-6, and EX-8 range from 95° to 96°, all of which have zero blank holding pressure. EX-2, EX-4, and EX-9 have fiber-enclosed angles around 87°, with a blank holding pressure of 1.18 kPa. When the blank holding pressure is increased to 2.36 kPa, the fiber-enclosed angle decreases to around 83°. Higher blank holding pressures indicate higher extension forces during the thermoforming process and result in fewer fiber-enclosed angles. Therefore, blank holding pressure is the most significant factor affecting the fiber-enclosed angle, and this angle decreases with an increase in blank holding pressure. On the other hand, the effects of blank temperature and mold temperature may be negligible. The overall results suggest that an appropriate blank holding pressure is around 1.18 kPa.

**Figure 9:**
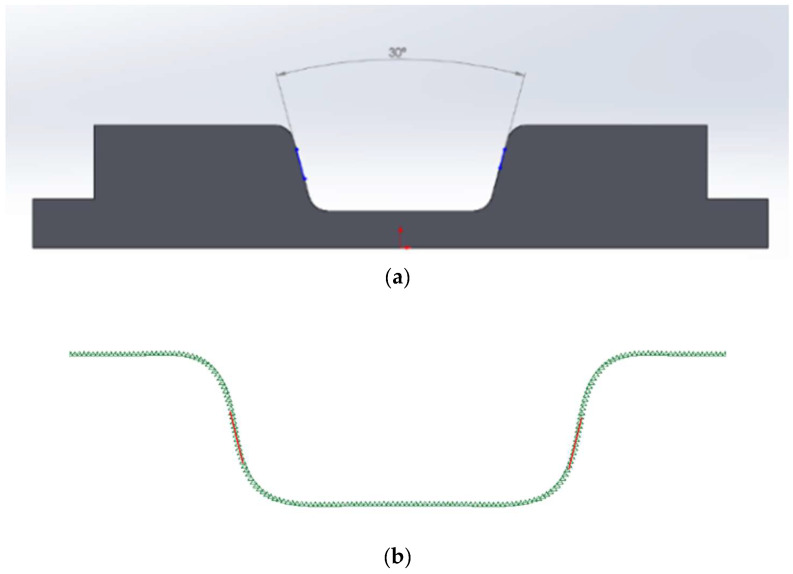
Angle on the mold and measure on the workpiece. (**a**) Mold angle; (**b**) Angle measured on the workpiece.

After thermoforming and demolding, the ideal enclosed angle between the two walls of the U-shaped structure, as shown in Figure 9, is 30°. If the angle is greater than 30°, it is called spring-back, defined as the difference between the final enclosed angle and the ideal angle. The spring-back angle is positive when spring-back occurs. If the angle is less than 30°, it is called spring-in, and the spring-back value is negative. The spring-back angles obtained from different combinations of process parameters are shown in Table 5. It is evident that EX-2, EX-3, EX-4, and EX-9 have minimal spring-back angles. In contrast, EX-5 and EX-7 exhibit large spring-back angles, possibly due to the high blank holding pressure causing outward extension. On the other hand, EX-1, EX-6, and EX-8 have negative angles, indicating spring-in, which may result from zero blank holding pressure causing inward contraction. The low spring-back angle of EX-3 may come from the high blank temperature. Thus, from the results of EX-2, EX-4, and EX-9, blank holding pressure is also a critical parameter for spring-back behavior, with an appropriate value around 1.18 kPa. The blank temperature may have an effect on the spring-back angle, while the effect of mold temperature may not be clear.

**Figure 10:**
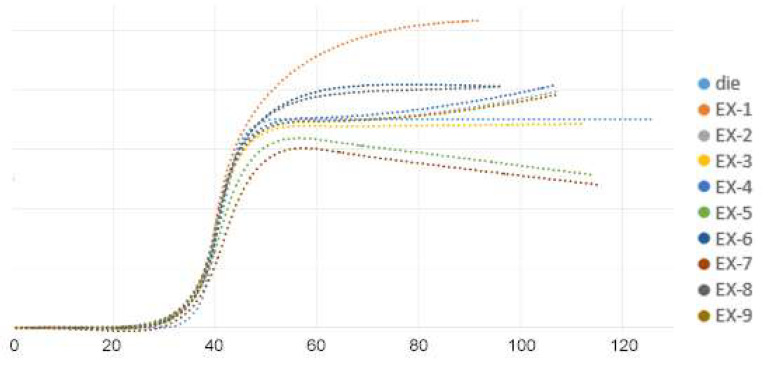
Profiles of the half-U-shaped structure after demolding.

The fitness of the workpiece is defined to measure the difference between the profile of the workpiece and the mold shape. The calculation of fitness is as follows:

(1) $$Fitness=\sqrt{{\sum (A_{i}-B_{i})}^{2}+{\left(A_{j}-B_{j}\right)}^{2}}$$

where *A_i_* and *A_j_* are the *x* and *y* coordinates of the workpiece after thermoforming, and *B_i_* and *B_j_* are the *x* and *y* coordinates of the corresponding point of the mold before thermoforming. A lower value of fitness indicates that the desired shape of the workpiece has been achieved. Figure 10 shows the final profiles of the half-workpieces after thermoforming with the parameter combinations from the Taguchi array. This figure clearly demonstrates that the spring-back behavior in EX-3, EX-5, and EX-7 results in the flange of the workpiece being lower than that of the mold. Similarly, due to zero blank holding pressure, the fitness values for EX-1, EX-6, and EX-8 are large. High blank holding pressure in cases like EX-3, EX-5, and EX-7 also resulted in poor fitness. The best fitness was observed in EX-2, where the blank holding pressure was 1.18 kPa, the blank temperature was 230 °C, and the mold temperature was 190 °C. Again, blank holding pressure plays the main role, while the two temperatures could influence fitness. Instead of a low temperture, it is better to have a blank temperature close to the melting point of this material.

**Figure 11:**
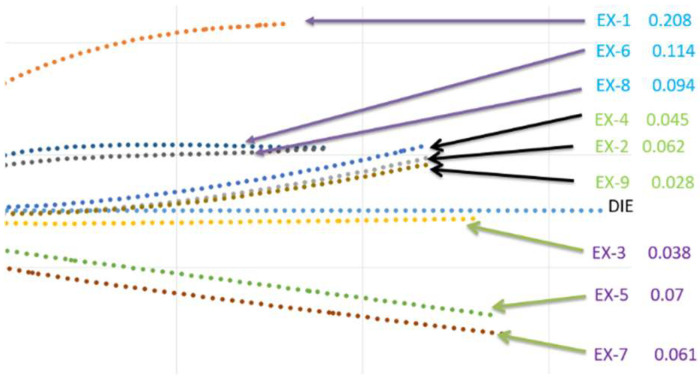
Strains of U-shaped structure after uniaxial loading.

The strain was defined as the length change of the workpiece divided by its original length when a 50 MPa uniaxial load was applied to one end while the other end was fixed. This strain was obtained in the third stage of analysis, following the thermoforming and demolding stages. The calculated strains under different combinations of the three parameters are shown in Table 5, and the profiles of the right half of the workpiece are shown in Figure 11, despite the lack of symmetry. Since strong structures are desired, lower strain values indicate better structural integrity. As shown in blue, EX-1, EX-6, and EX-8 have the largest strains compared to the other cases, which correlates with the trends seen in fitness and the fiber-enclosed angle, and these cases have zero blank holding pressure. The other six cases exhibit similar strain values. The three cases highlighted in purple—EX-3, EX-5, and EX-7—have higher fitness or original lengths but smaller fiber-enclosed angles. The three cases in green have intermediate values of fitness and fiber-enclosed angles. From the viewpoint of strain, EX-3 and EX-9 are the best combinations, with EX-2 and EX-5 also performing well. These results suggest a complicated combination of the three processing parameters. In addition, the use of blank holding pressure, higher blank temperatures, and lower mold temperatures are also suggested.

Considering the four quality results, including fiber-enclosed angle, spring-back angle, fitness, and strain, blank holding pressure has the most significant effect, blank temperature has some effect, and mold temperature has a minor effect. Additionally, these four quality values are interdependent and exhibit similar trends. They could be considered fiber-dominant quantities mainly affected by blank holding pressure. Comparing all the results from the Taguchi orthogonal array, EX-2 and EX-3 show very good outcomes. The best choice is EX-2. Therefore, the optimal combination of process parameters appears to be a blank holding pressure of 1.18 kPa, a blank temperature of 230 °C, and a mold temperature of 190 °C.

## 5. Conclusions

In this work, the effects of thermoforming parameters on thermoplastic composites consisting of woven carbon fiber fabric and polycarbonate were investigated using finite element simulation and the Taguchi orthogonal array. The considered thermoforming process parameters were blank temperature, mold temperature, and blank holding pressure, with three levels selected for each factor. The discrete finite element method, incorporating micro-mechanical models, was used to simulate the thermoforming, demolding, and loading processes. The objectives of the Taguchi array were to find optimal values for the fiber-enclosed angle, spring-back angle, fitness, and strain of the U-shaped workpiece. Verification of the discrete finite element method was performed by comparing stress-displacement curves obtained from bias extension tests at five different temperatures. When the fiber-enclosed angles were the objectives, results from the Taguchi orthogonal array indicated that the dominant parameter was the blank holding pressure, with the optimal value being 1.18 kPa, while the effects of the other two factors could be neglected. As for the spring-back angle, both positive and negative values were possible, and the blank holding pressure was the main factor, with a suitable value of 1.18 kPa. Similar trends were found for fitness and strain, encouraging the use of appropriate blank holding pressure, higher blank temperatures, and lower mold temperatures. Overall, blank holding pressure emerged as the dominant factor, with blank temperature having the second most significant effect and mold temperature having a minor effect. Additionally, the four quality values were interdependent and exhibited similar trends, suggesting that the best combination of process parameters is a blank holding pressure of 1.18 kPa, a blank temperature of 230 °C, and a mold temperature of 190 °C.

## Figures and Tables

**Table 1:** Some properties of carbon fiber.

Property	Value	Unit
Density	1.8	g/cm^3^
Longitudinal Young’s modulus	230	GPa
Transverse Young’s modulus	25.3	GPa
Shear modulus	12	GPa
Poisson’s ratio	0.24	
Heat capacity	720	J·°C^−1^
Longitudinal thermal conductivity	−0.18	W/m·K
Transverse thermal conductivity	0.18	W/m·K
Coefficient of thermal expansion	−1 × 10^−6^	1/°C
Ultimate strain at failure	0.03	
Yarn locking angle	5	Degree

**Table 2:** Some properties of polycarbonate.

Property	Value	Unit
Density	1.2	g/cm^3^
Young’s modulus	2.9	GPa
Poisson’s ratio	0.39	
Heat capacity	1250	J·°C^−1^
Thermal conductivity	2	W/m·K
Coefficient of thermal expansion	67 × 10^−6^	1/°C

**Table 3:** Taguchi orthogonal array.

Factor	Blank Temperature	Mold Temperature	Blank Holding Pressure
EX-1	230 °C	230 °C	0 kPa
EX-2	230 °C	190 °C	1.18 kPa
EX-3	230 °C	160 °C	2.36 kPa
EX-4	190 °C	230 °C	1.18 kPa
EX-5	190 °C	190 °C	2.36 kPa
EX-6	190 °C	160 °C	0 kPa
EX-7	160 °C	230 °C	2.36 kPa
EX-8	160 °C	190 °C	0 kPa
EX-9	160 °C	160 °C	1.18 kPa

**Table 4:** Fiber-enclosed angles.

	A	B	C	D	E	F	G	Ave.
EX-1	94.48°	96.44°	94.48°	96.44°	94.48°	96.44°	94.48°	95.32°
EX-2	95.04°	89.10°	81.16°	83.14°	81.16°	89.10°	95.04°	87.68°
EX-3	84.56°	84.56°	80.34°	83.16°	80.34°	84.56°	84.56°	83.16°
EX-4	95.16°	89.18°	81.20°	83.20°	81.20°	89.18°	95.16°	87.74°
EX-5	84.34°	84.34°	78.34°	81.34°	78.34°	84.34°	84.34°	82.18°
EX-6	95.82°	97.94°	93.58°	95.82°	95.82°	97.94°	95.82°	96.10°
EX-7	84.88°	84.8°8	80.34°	82.60°	80.34°	84.88°	84.88°	83.26°
EX-8	95.16°	97.20°	95.16°	95.16°	95.16°	97.20°	95.16°	96.02°
EX-9	95.22°	89.30°	81.40°	83.38°	81.40°	89.30°	95.22°	87.88°

**Table 5:** Fitness, spring-back angle, and strain.

	Spring-Back Angle	Fitness	Strain
EX-1	−3.65°	19.77	0.208
EX-2	−0.32°	2.00	0.062
EX-3	0.23°	3.41	0.038
EX-4	−0.08°	3.00	0.045
EX-5	4.61°	4.99	0.070
EX-6	−1.45°	9.44	0.114
EX-7	13.07°	6.44	0.061
EX-8	−2.60°	7.06	0.094
EX-9	0.43°	3.59	0.062

## Data Availability

Data are available upon request from the corresponding author.
